# Effects of multicomponent primary care-based intervention on immunization rates and missed opportunities to vaccinate adults

**DOI:** 10.1186/s12875-020-01115-y

**Published:** 2020-02-29

**Authors:** Natalia Y. Loskutova, Craig Smail, Elisabeth Callen, Elizabeth W. Staton, Niaman Nazir, Brian Webster, Wilson D. Pace

**Affiliations:** 1 American Academy of Family Physicians National Research Network, 11400 Tomahawk Creek Pkwy, Leawood, KS 66211 USA; 2 Department of Family Medicine, University of Colorado, Mail Stop F496, 12631 E. 17th Ave, Room 3403, Aurora, CO 80045 USA; 3 Department of Preventive Medicine and Public Health, University of Kansas Medical Center, Mailstop 1008, 3901 Rainbow Blvd, Kansas City, KS 66160 USA; 4 Wilmington Health, 1202 Medical Center Dr, Wilmington, NC 28401 USA; 5 DARTNet Institute, 12635 East Montview Blvd, Suite 127, Aurora, CO 80045 USA

**Keywords:** Primary care, Immunization, Adult, Multi-component interventions

## Abstract

**Background:**

Adult immunization rates are below *Healthy People 2020* targets. Our objective was to evaluate the effectiveness of a multicomponent intervention to improve adult immunization rates.

**Methods:**

This prospective interventional before-and-after non-randomized study was conducted through the American Academy of Family Physicians National Research Network with 43 primary care physicians from a large multi-specialty healthcare organization (multicomponent intervention group *n* = 23; comparator group *n* = 20) in the United States. The multicomponent intervention included provider reminders, quarterly provider-level performance reports, provider education, patient visual aid materials, and standing orders on adult pneumococcal, influenza, and zoster immunizations. We assessed individual and comparative provider-level vaccination rates and missed opportunities detailing concordance with targets established by Healthy People 2020 for pneumococcal, influenza, and zoster immunizations.

**Results:**

Vaccination rates increased after 12 months in intervention and comparator groups respectively for: a). influenza from 44.4 ± 16.7 to 51.3% ± 12.9% (by 6.9 percentage points, *p* = 0.001) and from 35.1 ± 19.1 to 41.3% ± 14.2%, (by 6.2 percentage points, *p* = 0.01); b). pneumococcal vaccinations in older adults from 62.8 ± 17.6 to 81.4% ± 16.6% (by 18.6 percentage points, for *p* < 0.0001) and from 55.9 ± 20.0 to 72.7% ± 18.4% (by 16.7 percentage points, *p* < 0.0001); and c). zoster from 37.1 ± 13.4 to 41.9% ± 13.1% (by 4.8 percentage points, *p* < 0.0001) and from 35.0 ± 18.7 to 42.3% ± 20.9% (7.3 percentage points, *p* = 0.001). Pneumococcal vaccinations in adults at risk did not change from baseline in intervention group (35.7 ± 19.6 to 34.5% ± 19.0%, *p* = 0.3) and improved slightly in comparator group (24.3 ± 20.1 to 28.2% ± 20.0%, *p* = 0.003). Missed opportunities reduced after 12 months, most noticeably, for: a). for influenza from 57.7 to 48.6% (by 9.1 percentage points, *p* < 0.0001) and from 69.7 to 59.6% (by 10.1 percentage points, *p* < 0.0001); b). pneumococcal vaccinations in older adults from 18.1 to 11.5% (by 6.6 percentage points *p* < 0.0001) and from 24.6 to 20.4% (by 4.3 percentage points, *p* < 0.0001) in intervention and comparator groups respectively.

**Conclusions:**

Multicomponent interventions show promise in improving vaccination rates and reducing missed opportunities in older adults for pneumococcal and zoster vaccines and vaccination against influenza. Provider reminders remain the most effective strategy when delivered either as a component of these interventions or alone.

## Background

Adult immunization rates remain well below *Healthy People 2020* targets, [1] even though adults, on average, visit a primary care provider more than three times a year [2]. Primary care physicians provide more than 560 million office visits each year, putting them in the unique position to administer immunizations to patients of all ages [3]. Specialty care provides more than 230 million office visits, but, based on low immunization rates in specialty care, a large proportion of these visits are viewed as missed opportunities to provide adequate vaccination for adults [4].

We recently explored the missed opportunities in adult vaccinations in a meta-narrative literature review and concluded that missed opportunities are present in various health care settings [5]. Moreover, we reported two methodologies for quantifying missed opportunities: 1. Based on the number of healthcare encounters without appropriate vaccination services, defined as a number or percentage of visits per patient with no vaccination related services (missed opportunities per patient or patient-level); and 2. Based on vaccination status as “non-vaccinated” among a group of patients who had a health care encounter where the vaccination should/could have happened, presented as percentage of non-vaccinated patients who had an encounter with health care providers (missed opportunities per patient population or population-level) [5]. We suggested that missed opportunities could represent a more accurate measure of provider and health care organization performance as compared to population-wide immunization rates because missed opportunities are clearly associated with a patient encounter.

The low vaccination rates among adults, and especially among adults age 19–64 years, [6] are not surprising as the vaccination recommendations are complicated by revaccination recommendations and strategies, the varying target immunization ages and goals, the current immunization rates that differ for high-risk adults and elderly, and the annual changes to the vaccination schedules. Even though the recommendations are reviewed, endorsed, and promoted by several major medical professional organizations and CDC, [7] providers and patients encounter many barriers, and the efforts still result in low vaccination rates among adults [8, 9].

A comprehensive report by Task Force on Community Preventative Services (Task Force) recommends several interventions with strong evidence for their effectiveness in improving immunization rates. Of all interventions used alone, provider reminders have sufficient evidence of effectiveness to be currently recommended. The Task Force recommends that interventions implemented in combination (multicomponent interventions) should be considered for improving vaccination coverage among high-risk populations; specifically, the multicomponent interventions that combine one or more interventions from three categories: 1. enhancing access to care and reducing administrative barriers; 2. implementing provider and system-based interventions such as provider reminders; and 3. increasing vaccination demand among patients have been shown to increase immunizations rates [10].

The purpose of this paper is to present the results of a project that tested a multicomponent approach to increase immunization rates and reduce missed opportunities in adults. Based on our earlier work, we also aimed to test the missed opportunities as an outcome of the multicomponent intervention in addition to standard metrics for vaccination rates. The project aimed to test the generalizability of the Task Force recommendations to primary healthcare settings and different patient populations.

## Methods

### Study design

This was a prospective interventional before-and-after non-randomized study with an intervention arm and a comparator arm to evaluate the effectiveness of a multicomponent intervention to improve immunizations in a multispecialty medical group. The primary care providers willing to receive a multicomponent intervention were allocated to the intervention group, the rest of the primary care providers elected to participate in the comparator group. This was a pragmatic trial with no direct patient recruitment or enrollment. This study was conducted as quality improvement project between July, 2015, and August, 2016, where the intervention period encompassed at least one full influenza season. The intervention targeted the organization for system-level changes and individual providers for improving physician behaviors and patient care. The comparator group received a single provider-level intervention in the form of point-of-care reminders, and the intervention group received multicomponent intervention as described below. The American Academy of Family Physicians Institutional Review Board (AAFP IRB) approved the study.

### Setting and participants

The study was conducted in one large health care organization in North Carolina, USA, and focused on general internal medicine and family medicine settings. The organization is a multi-specialty clinic (including pulmonology, infectious disease, family medicine, and internal medicine) with primary care providers integrated into the system. From ten sites 43 providers within the same organization were included in the study; 23 primary care providers participated in the intervention arm of the study while 20 providers were in the comparator group. The professional composition of the intervention and comparator groups respectively included: physician assistants (1 in each group); nurse practitioners (2 and 1); family practice physicians (4 and 12); and general internal medicine physicians (16 and 6).

### Patient population overview

This study included all patients age 18 years and older receiving services at the participating providers throughout the year and during flu season from 2013 through 2015 who were eligible for pneumococcal and influenza vaccination. The patient cohorts were defined as number of unique eligible patients among all adults in each of the subgroups:

- Pneumococcal:
  - all age 65 and older
  - age 19–64 with at least one risk factor (see Appendix 1 for a list of included conditions)
- Influenza: All age 18 and older
- Zoster: all age 60 and older

Eligible patients were included if they had at least one visit to any primary care provider from the list of participating providers within a timeframe of data analysis (year, season, or a specific time period) and further assigned to a provider based on the majority rule (i.e., patients assigned to the provider they saw the most for a given time period).

### Key elements of intervention

#### Clinical decision support (CDS) provider reminders

The project team delivered this component to the providers in both intervention and comparator groups. The project team developed or updated algorithms for CDS Order Sets for provider reminders and standing orders for adult vaccinations according to the current detailed guidelines for vaccination and revaccination of adult patients. The final algorithms that supported the CDS systems developed by the study team with the input from the lead clinician are available from the authors.

Each provider in both the intervention and comparator groups was able to view a patient’s recommendation report at the time of the visit. The report included action items for the provider and staff based on current vaccination eligibility, recommendations, patient immunization history, and status at the time of the visit. The system was distributed for all primary care providers to use for the duration of the study.

The following components were delivered to providers in the intervention group only.

#### Standing orders

At the beginning of the study, the participant organization did not systematically use standing orders. For adult vaccines, three providers in the intervention group reported always having standing orders, nine providers reported sometimes having standing orders; while six providers reported occasionally or never having standing orders. Educational materials on standing orders were developed for the influenza and pneumococcal vaccinations to encourage the providers, practice staff, and leaders to consider adopting standing orders. At the beginning of the project, the organization received these practice education materials and implementation strategies (available from the authors).

#### Provider audit and feedback

The project team developed quarterly provider-level and overall reports using practice’s electronic health records data. Providers and staff met quarterly to review and discuss provider-level and overall reports. The providers were identified by the name in the reports and whenever necessary, peer pressure or alternative performance improvement strategies (ranking, positive reinforcement, competition, incentives etc.) were applied. Clear performance goals for reducing missed opportunities for vaccination at every visit were emphasized.

#### Improving documentation

This intervention component consisted of technical training for providers that focused on improving documentation in two areas: 1. Past immunization history – asking patients about their recent vaccinations and documenting in the EHR; and 2. Documenting current immunization status with focus on vaccinations offered at the visit (record of vaccination offered and given or declined by patients).

#### Provider education and communications

Providers received materials for patient and staff immunization education, talking points for communication with patients about immunizations, technical guidelines on vaccine storage and billing/coding recommendations, recommendations on how to address vaccine shortages, and guidance on expanding access to vaccination services. A monthly newsletter about the project was produced and shared with the participants.

#### Enhancing patient outreach

The project incorporated the following strategies in patient education and outreach to increase demand and reduce refusals: educating physicians and their practice staff about effective patient engagement; increasing patient awareness and acceptance of recommended vaccines; and patient education during the visits via visual aids. A set of patient-facing educational materials developed by the CDC and other sources was provided to the site.

### Data collection and evaluation

#### Data sources

The data for the study were obtained from the EHR. The overall total number of visits was collected for each provider. For patients who visited more than one provider in a given time period, these patients were assigned to the provider they saw the most (which we called the majority-rule approach). For patients, we extracted demographics, current diagnoses, smoking status, and vaccines given, discussed, or refused with reasons for refusal whenever possible. Records on vaccines given elsewhere were obtained from historic records as documented in EHR. The data were collected every three months and summarized for immunization rates for provider feedback, and then used for longitudinal data analysis. Vaccination rates are presented here as percentage of vaccinated individuals over all eligible persons for a given vaccine within the timeframe reported. We established baseline immunization rates via EHR data pulls by reviewing immunization performance data for patients seen in the year or influenza season prior to the beginning of the intervention. The definitions for missed opportunities were based on operational methodologies developed by the project team and included population-based, patient/visit-based, and reminder-based approaches [5].

Briefly, in the population-based missed opportunity metric, we defined missed opportunity as the percentage of eligible unvaccinated patients with at least one scheduled visit where the vaccination should/could have happened over the observation period (one year or one season as applicable).

In the visit-based metric or missed opportunities per patient (patient-level) we defined missed opportunity based on the number of healthcare encounters without appropriate vaccination services, defined as a number of visits per patient with no vaccination related services. We counted the number of visits for all eligible patients (i.e., those matching cohort criteria, matching to one of the participating providers by majority-rule) as well as the number of visits where a provider is compliant (vaccine given or vaccine offered but refused). Further, we presented these results clustered by the patient’s primary provider using the majority-rule approach. A visit was considered a missed opportunity if a vaccination record or vaccine order that was refused by the patient did not appear either on or within 7 days after the date of visit to account for possible delays with data entry.

### Statistical analyses

Discrete variables are described using frequency and percentage. Means and standard deviations (SD) are used to describe continuous variables. T-tests were used for comparing groups in case of continuous variables – for before and after the intervention period the paired samples t-test were used, and independent sample t-tests were used to assess unadjusted differences between the intervention and comparator groups. Hierarchical linear regressions were also used to examine the relationship between vaccination rates (dependent variable), and predictor variables: group assignment (intervention or comparator) and baseline vaccination rates. Pearson correlations between the vaccination rates and the missed opportunity including population-based and visit-based metric were also calculated. Statistically significant associations and differences were identified by *p*-values of less than 0.05. All analyses were conducted using SAS version 9.4 (copyright 2002–2012 by SAS Institute Inc., Cary, NC, USA).

### Role of the funding source

This work was supported in part by a research grant from Investigator-Initiated Studies Program of Merck Sharp & Dohme Corp. Merck Sharp & Dohme Corp. was not involved in the study design, data collection, data analysis and interpretation, writing or reporting of this work, and did not have any involvement in the decision to submit this article for publication. The opinions expressed in this paper are those of the authors and do not necessarily represent those of Merck Sharp & Dohme Corp.

## Results

### Participant characteristics

Forty-four providers were initially included in the study. One provider left the practice shortly after enrollment. There were 23 clinicians in the intervention group (four family medicine physicians, sixteen internal medicine physicians, two nurse practitioners, and one physician assistant). The comparator group (*n* = 20) included twelve family medicine physicians, six internal medicine physicians, one nurse practitioner, and one physician assistant. Patient characteristics for all unique patients are presented in Table 1.

**Table 1:** Overview of included patient cohorts seen by participating study providers

Year	Unique adult patients seen by providers in the intervention group (n)	Unique adult patients seen by providers in the comparator group (n)	Total Patient Sample			
			Unique patients (n)	Gender		Age (mean ± SD)
				Male (n,%)	Female (n,%)	
Calendar year:						
2013	18,244	12,577	67,993	26,337 (38%)	41,656 (62%)	54 ± 18.2
2014	27,415	21,465	110,617	44,028 (39%)	66,589 (61%)	52 ± 18.7
2015	30,844	24,906	108,240	42,312 (39%)	65,928 (61%)	53 ± 18.1
Flu season:						
2013–14	20,952	15,076	77,985	30,535 (39%)	47,450 (61%)	53 ± 18.2
2014–15	24,506	17,256	82,464	31,960 (38%)	50,504 (62%)	53 ± 18.1
2015–16	26,226	20,356	87,931	34,024 (38%)	53,907 (62%)	54 ± 17.7

Calendar year eligibility: A patient is considered to be eligible for the cohort each year if they (1.) are 18 years of age and older as of first day of the year and (2.) they had > = 1 encounter at some point during the year

Flu season eligibility: A patient is considered to be eligible for the cohort each season if they (1.) are 1818 years of age and older as of first day of the flu season and (2.) they had > = 1 encounter at some point during the flu season. In this study a flu season runs from 1-Sep to 31-Mar.

### Effects of intervention on vaccination rates

Baseline and end-of-study vaccination rates are presented in Table 2. Vaccination rates increased after 12 months in intervention and comparator groups respectively for: a). influenza from 44.4 ± 16.7 to 51.3% ± 12.9% (by 6.9 percentage points, *p* = 0.001) and from 35.1 ± 19.1 to 41.3% ± 14.2%, (by 6.2 percentage points, *p* = 0.01); b). pneumococcal vaccinations in older adults from 62.8 ± 17.6 to 81.4% ± 16.6% (by 18.6 percentage points, *p* < 0.0001) and from 55.9 ± 20.0 to 72.7% ± 18.4% (by 16.7 percentage points, *p* < 0.0001); and c). zoster from 37.1 ± 13.4 to 41.9% ± 13.1% (by 4.8 percentage points, *p* < 0.0001) and from 35.0 ± 18.7 to 42.3% ± 20.9% (by 7.3 percentage points, *p* = 0.001). Pneumococcal vaccinations in adults at risk did not change from baseline in intervention group (35.7 ± 19.6 to 34.5% ± 19.0%, *p* = 0.3) and improved slightly in comparator group (24.3 ± 20.1 to 28.2% ± 20.0%, 3.8 percentage point increase, *p* = 0.003). This difference for adults at risk was the only significant difference in percentage point changes between the intervention and comparator groups in (*p* = 0.001).

**Table 2:** Baseline and end of project vaccination rates

	Intervention (*n* = 23), mean ± SD	Comparator (*n* = 20), mean ± SD	*P*-value	HP 2020 Intervention (n*)	HP2020 Comparator (n*)
Y1 Influenza	44.4% ± 16.7%	35.1% ± 19.1%	0.096	1	0
Y2 Influenza	51.3% ± 12.9%	41.3% ± 14.3%	0.019	1	1
Y1 Pneumococcal (Age)	62.8% ± 17.7%	55.9% ± 19.0%	0.223	8	1
Y2 Pneumococcal (Age)	81.4% ± 16.6%	72.6% ± 18.4%	0.106	14	9
Y1 Pneumococcal (Risk)	35.7% ± 19.4%	24.3% ± 20.1%	0.066	3	3
Y2 Pneumococcal (Risk)	34.5% ± 19.0%	28.2% ± 20.0%	0.0295	3	4
Y1 Zoster	37.1% ± 13.4%	35.0% ± 18.7%	0.671	19	11
Y2 Zoster	41.9% ± 13.1%	42.3% ± 20.9%	0.940	19	11

Y1- baseline vaccination rates; Y2 - end-of-study vaccination rates; HP2020 - Healthy People 2020; n* - number of providers who reached HP2020 target by the end of intervention period; *p*-value for independent samples t-test of difference between groups at each time point, unadjusted for baseline rates

Regression models were used to examine the relationship between vaccination rates (dependent variable), and predictor variables: group assignment (intervention or comparator) and baseline vaccination rates (Table 3). Baseline vaccination rates had significant (*p* < 0.0001) zero-order associations with end of project vaccination rates for all cohorts. The intervention group assignment had statistically significant associations with improved vaccination rates over time for pneumococcal vaccinations in adults at risk (Group: *p* = 0.006; pneumococcal baseline vaccine rate: < 0.0001). The was no effect of intervention on rates for influenza vaccinations (Group: *p* = 0.080; influenza baseline vaccine rate: *p* = < 0.0001), pneumococcal vaccine in older adults (Group: *p* = 0.212; pneumococcal baseline vaccine rate: < 0.0001), and zoster vaccinations (Group: *p* = 0.174; zoster baseline vaccine rate: < 0.0001).

**Table 3:** Hierarchical Linear Regression Results

Variable	Standardized Coefficients	*p*-value	Level Statistics
Influenza			
Level 1			
Constant		< 0.0001	Adjusted R^2^: 0.105 Regression ANOVA *p*-value: 0.019
Group	0.355	0.019	
Level 2			
Constant		< 0.0001	Adjusted R^2^: 0.751 Regression ANOVA *p*-value: < 0.0001
Group	0.143	0.080	
Baseline rate	0.825	< 0.0001	
Pneumococcal (Age)			
Level 1			
Constant		< 0.0001	Adjusted R^2^: 0.040 Regression ANOVA *p*-value: 0.106
Group	0.250	0.106	
Level 2			
Constant		< 0.0001	Adjusted R^2^: 0.846 Regression ANOVA *p*-value: < 0.0001
Group	0.078	0.212	
Baseline rate	0.906	< 0.0001	
Pneumococcal (Risk)			
Level 1			
Constant		< 0.0001	Adjusted R^2^: 0.003 Regression ANOVA *p*-value: 0.295
Group	0.164	0.295	
Level 2			
Constant		0.002	Adjusted R^2^: 0.936 Regression ANOVA *p*-value: < 0.0001
Group	−0.118	0.006	
Baseline rate	0.996	< 0.0001	
Zoster			
Level 1			
Constant		< 0.0001	Adjusted R^2^: 0.000 Regression ANOVA *p*-value: 0.940
Group	−0.012	0.940	
Level 2			
Constant		0.005	Adjusted R^2^: 0.879 Regression ANOVA *p*-value: < 0.0001
Group	−0.075	0.174	
Baseline rate	0.943	< 0.0001	

We did not conduct subgroup analyses for vaccination rates and changes over time among individual risk factor sub-groups of patients age 19–64 years with various risks for pneumococcal disease. This was due to a small sample size for several conditions and absence of main effects in overall change in vaccination rates in this group of patients.

### Missed opportunities analyses

The Tables 4 and 5 show the rates of missed opportunities across the four study vaccination cohorts using two missed opportunity metrics for influenza, pneumococcal (age), pneumococcal (risk), and zoster.

**Table 4:** Missed Opportunities Before and After Intervention: Population-level Metric

Provider	2014–15		2015–16		p-value
Influenza	N	Missed Opportunity	N	Missed Opportunity	
Intervention (*n* = 23)	23,630	57.7%	23,976	48.6%	0.000
Comparator (*n* = 20)	17,970	69.7%	19,173	59.6%	0.000
Overall (*n* = 43)	41,600	62.9%	43,149	53.5%	0.000
Pneumococcal (Risk)					
Intervention (*n* = 23)	5066	60.8%	5509	60.3%	0.604
Comparator (*n* = 20)	3042	70.4%	3730	69.5%	0.453
Overall (*n* = 43)	8108	64.4%	9239	64.0%	0.612
Pneumococcal (Age)					
Intervention (*n* = 23)	8689	18.1%	9148	11.5%	0.000
Comparator (*n* = 20)	5422	24.6%	5965	20.4%	0.000
Overall (*n* = 43)	14,111	20.6%	15,113	15.0%	0.000
Zoster					
Intervention (*n* = 23)	11,991	55.3%	9987	49.7%	0.000
Comparator (*n* = 20)	7379	53.6%	6453	52.7%	0.298
Overall (*n* = 43)	19,370	54.7%	16,440	50.9%	0.000

N = total number of eligible patients with at least one visit

Date ranges:

2014–15 = 1st September 2014 - 31st August 2015

2015–16 = 1st September 2015 - 31st August 2016

**Table 5:** Missed Opportunities Before and After Intervention: Visit-Based Metric

Provider	2014–15		2015–16		p-value
Influenza	N	Missed Opportunity	N	Missed Opportunity	
Intervention (n = 23)	315,147	31.5%	333,126	39.1%	0.000
Comparator (n = 20)	214,742	42.0%	244,137	50.6%	0.000
Overall (n = 43)	529,889	35.8%	577,263	43.9%	0.000
Pneumococcal (Risk)					
Intervention (n = 23)	460,353	28.5%	722,514	31.8%	0.000
Comparator (n = 20)	210,447	40.8%	316,897	47.3%	0.000
Overall (n = 43)	670,800	32.4%	1,039,411	36.5%	0.000
Pneumococcal (Age)					
Intervention (n = 23)	140,255	8.1%	151,162	8.2%	0.136
Comparator (n = 20)	89,045	12.4%	97,075	14.9%	0.000
Overall (n = 43)	229,300	9.7%	248,237	10.8%	0.000
Zoster					
Intervention (n = 23)	184,815	52.3%	197,482	51.6%	0.000
Comparator (n = 20)	112,128	49.7%	125,321	52.1%	0.000
Overall (n = 43)	296,943	51.3	322,803	51.8%	0.000

N = total number of eligible patients with at least one visit

Date ranges:

2014–15 = 1st September 2014 - 31st August 2015

2015–16 = 1st September 2015 - 31st August 2016

#### Population-based missed opportunities

Missed opportunities reduced after 12 months for: a). influenza from 57.7 to 48.6% (by 9.1 percentage points, *p* < 0.0001) and from 69.7 to 59.6% (by 10.1 percentage points, *p* < 0.0001); b). pneumococcal vaccinations in older adults from 18.1 to 11.5% (by 6.6 percentage points *p* < 0.0001) and from 24.6 to 20.4% (by 4.3 percentage points, *p* < 0.0001) in intervention and comparator groups respectively. Missed opportunities for zoster vaccinations decreased in the intervention group from 55.3 to 49.7% (by 5.6 percentage points *p* < 0.0001) and did not change in the comparator group (53.6% vs. 52.7%, 0.9 percentage points difference, *p* = 0.3).

Missed opportunities in pneumococcal vaccinations in adults at risk did not change in either group (Table 4).

#### Patient- (visit-) based missed opportunities

The total numbers of visits provided by all providers in the study defined as vaccination eligible visits and visit-based missed opportunities are presented in Table 5. Visit-based missed opportunities were reduced significantly only for zoster vaccinations in the intervention group (55.3% vs. 49.7%, *p* < 0.0001). No other reductions in missed opportunity visits were observed in either group.

### Correlations between vaccination rates and missed opportunities

As shown in Table 6, the missed opportunities metrics generated by two primary methods (population and patient/visit) correlated significantly with vaccination rates at both baseline and end of study. Lower vaccination rates were strongly associated with more missed opportunities that was consistently observed for both missed opportunity metrics.

**Table 6:** Correlations Between Vaccination Rates and Missed Opportunities

Vaccination Rates	Intervention		Comparator	
	Missed opportunities population-based metric	Missed opportunity visit-based metric	Missed opportunities population-based metric	Missed opportunity visit-based metric
Y1 Influenza	−0.99**	−0.93**	−0.94**	− 0.91**
Y1 Pneumococcal (Age)	−0.93**	− 0.84**	− 0.86**	− 0.83**
Y1 Pneumococcal (Risk)	− 0.97**	− 0.93**	−0.95**	− 0.72**
Y1 Zoster	−0.97**	− 0.97**	− 0.89**	−0.87**
Y2 Influenza	−0.99**	−0.98**	− 0.94**	−0.93**
Y2 Pneumococcal (Age)	−0.99**	−0.99**	− 0.97**	−0.98**
Y2 Pneumococcal (Risk)	−0.98**	−0.93**	− 0.98**	−0.91**
Y2 Zoster	−0.96**	−0.97**	− 0.97**	−0.96**

**Correlation significant at *p* < 0.01; Y1 - baseline vaccination rates; Y2 - end-of-study vaccination rates; inverse association (−) indicates that lower vaccination rates correlated with more missed opportunities

## Discussion

Despite long-standing recommendations to vaccinate adults against vaccine-preventable diseases such as influenza, pneumococcal disease, and shingles, the rates of vaccination coverage among adults remain low. The vaccination rates in this study were comparable to the national benchmarks [6] at the beginning of the study, and afterward exceeded the national rates in both groups, on all vaccines. However, not every provider was able to reach vaccination targets set by the *Healthy People 2020*, which indicates that the likelihood of receiving recommended vaccination may be more provider-dependent than previously thought, and having all providers meet the *Healthy People 2020* targets may require additional approaches and perhaps more time to see noticeable change [11].

This study demonstrated improvements in vaccination rates in both intervention group by 18% and the comparator group by 16% for pneumococcal vaccines in older adults and smaller improvements in influenza and zoster vaccination. The results for the comparator group that received only CDS provider reminders is comparable to rates reported by other studies for the same intervention when used alone [10]. The intervention groups received a combination of interventions, and overall demonstrated improvements moderately but not significantly beyond those seen in the comparator group. In the pneumococcal vaccinations for adults19–64 years old at risk, however, the multicomponent intervention showed no increase in vaccination rates or reductions in missed opportunities. Future studies need to consider the balance of impact vs. costs of multicomponent interventions compared to CDS when used alone.

The pneumococcal vaccine rates among the adults aged 19–64 years with risk factors did not change considerably and remained substantially below *Healthy People 2020* targets in both groups. Why the rates did not change in response to intervention remains unclear. We expect that despite the National Vaccine Advisory Committee recommendations, [12] providers may be hesitant to make strong recommendations for needed vaccines for adults aged 19 to 64 years. Future studies need to focus on exploring barriers to vaccinate in this particularly challenging group and consider interventions to maximize the value of individual intervention components that are effective in this patient population.

Our study demonstrated improvements in both provider groups that received the CDS point-of-care reminders. Our study corroborates previous reports that provider reminders remain the most effective way to increase vaccination rates at the provider level [13]. Studies exploring impact of CDS in general practice, however, indicate substantial levels of provider “alert fatigue,” and future studies need to explore ways to keep providers engaged in continuous vaccination improvement efforts [14, 15]. Additionally, in order for the CDS systems to be most effective, they have to rely on accurate evidence-based clinical algorithms that need to be regularly updated and aligned among multiple systems, settings, and patient populations. Future efforts need to be taken in designing CDS systems or a combination of interventions that improve attention to the at-risk adult group, as the existing CDS or a combination of interventions seem to have low effectiveness in this group [16].

Significant missed opportunities have been identified in both groups using at least two metrics – population-based and patient/visit-based, suggesting need for further understanding and research on how to reduce missed opportunities. Similar to the vaccination rates, however, no changes in missed opportunities for pneumococcal vaccination were observed in the adults aged 19–64 years at risk. The reasons for low intervention effects on any outcomes related to this group need further explorations, as it seems that the CDS has modest effect on the rates of vaccination in this group among providers with low vaccination rates, and the additional components of the intervention did not improve the rate any further. The intervention has been effective in reducing population-based missed opportunities; however, the overwhelming majority of patients who were still not vaccinated by the end of study had at least one encounter with the providers over the study period. While the reasons for existing missed opportunities need to be explored in future studies, the high level of correlations among vaccination rates and missed opportunities suggest that low vaccination rates among those who visited the clinic/provider in the reporting period can, once again, be explained primarily by provider-related factors that result in missed opportunities.

The missed opportunities methodology needs further research, in particular the visit-based metric. As the numbers of visits to primary care providers increase every year, the methodology needs to account for total number of visits in the study period as well as the number of eligible individuals. While we believe the percentage approach to this metric is reliable, it does not demonstrate the actionable reduction in the actual number of missed opportunities visits due to intervention.

The study had several limitations, including non-randomized study design with existing baseline vaccination level differences between the groups. Although we accounted for these differences statistically through regression analyses, it is possible that the providers who volunteered to test the multicomponent intervention placed a higher priority on vaccinations and were more successful in vaccinating their adult patients than those who did not volunteer. We have not explored the effects of professional group compositions or the effects of office affiliation/location/culture, which may have contributed to the baseline and overall vaccination rate and missed opportunity differences. The 16.7% older adult pneumococcal vaccination rate increase in the comparator group, however, demonstrates that CDS as a single intervention may be as effective in the group of providers who did not volunteer to receive other invention components. We used a “majority rule approach” that retrospectively assigned patients to a primary care provider based on the highest proportion of patient visits during each observation period. While this method has general limitations [17], the majority of patents in our study saw the same provider at least 75% of the time, and the rest fluctuated at random. Due to the relatively short duration, the study was limited in its ability to capture uptake and impact of standing orders because upon the study enrollment only three providers in the intervention group reported always having standing orders for adult vaccines, and shortly after the standing orders educational materials were delivered to the practice, the organization decided to roll out standing orders as an organization-wide quality improvement initiative. Large organization-wide quality improvement initiatives typically take long time to implement, and the standing orders were not fully adopted by all primary care providers by the conclusion of the study. This observation also demonstrates the unique challenges of implementing efficacious interventions from clinical trials in real world clinical settings where factors beyond researchers’ control may influence the fidelity of research. Additionally, we did not determine to what extent the participating providers shared practice staff who may have a role in vaccinations or to what extent that could have affected the study outcomes. Although most components of the multicomponent intervention, including CDS, targeted providers only, it is possible that some educational materials or patient-facing visual aids were accessible for practice staff.

## Conclusions

Primary care providers play a key role in delivering adult vaccinations. Despite multiple efforts to increase vaccination rates in adults, the vaccination coverage remains suboptimal, and significant missed opportunities still exist. Strong correlations between vaccination rates and missed opportunities to vaccinate patients at the time of the primary care visit suggest provider-related factors may be responsible.

Provider reminders remain the most effective intervention for vaccination rate improvements, but even they do not substantially reduce missed opportunities or facilitate substantial progress toward Healthy People 2020 adult immunization targets. While promising, various types and intensities of multicomponent intervention need to be further studied to maximize their impact on improving vaccination rates and reducing missed opportunities for vaccinating adults.

## Supplementary information

**Additional file 1.** Appendix 1 Patient Population Overview.

## Data Availability

The datasets generated or analyzed during the current study are not publicly available due to existing data sharing and use agreements and organizational policies in place but are available from the corresponding author on reasonable request.

## Abbreviations

SD: Standard deviation
