# Supplementary material for: Effects of multicomponent primary care-based intervention on immunization rates and missed opportunities to vaccinate adults
Source: BMC Fam Pract. 2020 Feb 29;21:46. doi: 10.1186/s12875-020-01115-y (PMC7049385; doi:10.1186/s12875-020-01115-y)
Supplement: Supplementary file 1 — Additional file 1. Appendix 1 Patient Population Overview. [file 12875_2020_1115_MOESM1_ESM.docx]

Appendix 1. Patient Population Overview

**Risk factor** - for pneumococcal vaccine - patients 19-64 are eligible/included when they have at least one of the following by the time of the visit based in the ICD-9 codes and/or corresponding ICD-10 codes:

*Risk Group 1*

- Asthma (493.X)

• Diabetes (250.X; 648.00-648.04)

• COPD (496.X; 492.8; 491.20-21; 494.0-494.1)

• Heart Failure (402.X; 404.X; 428.X) 'V45.81', 'V45.82'

• CAD (410.X-414.X)

- Current Smoker (305.1)

Risk Group 2 *(immunocompromised)*

• Renal Failure (585.00-586.99)

• Sickle Cell Dz (282.60-282.69)

• Asplenia (759.0-759.09)

• HIV (042.X)

• Cancer (203.00-208.92)
